# Measuring lung mechanics of expiratory tidal breathing with non-invasive breath occlusion

**DOI:** 10.1186/s12938-020-00777-0

**Published:** 2020-05-14

**Authors:** Sarah L. Howe, Melanie März, Sabine Krüger-Ziolek, Bernhard Laufer, Chris Pretty, Geoffery M. Shaw, Thomas Desaive, Knut Möller, J. Geoffrey Chase

**Affiliations:** 1 Department of Mechanical Engineering, University of Canterbury, Christchurch, New Zealand; 2 Institute of Technical Medicine (ITeM), Furtwangen University, Villingen-Schwenningen, Germany; 3 Department of Intensive Care, Christchurch Hospital, Christchurch, New Zealand; 4 GIGA Cardiovascular Science, University of Liege, Liege, Belgium

**Keywords:** Spirometry, Model-based methods, Expiration, Lung mechanics, Occlusion

## Abstract

**Background and objective:**

Lung mechanics measurements provide clinically useful information about disease progression and lung health. Currently, there are no commonly practiced methods to non-invasively measure both resistive and elastic lung mechanics during tidal breathing, preventing the important information provided by lung mechanics from being utilised. This study presents a novel method to easily assess lung mechanics of spontaneously breathing subjects using a dynamic elastance, single-compartment lung model.

**Methods:**

A spirometer with a built-in shutter was used to occlude expiration during tidal breathing, creating exponentially decaying flow when the shutter re-opened. The lung mechanics measured were respiratory system elastance and resistance, separated from the exponentially decaying flow, and interrupter resistance calculated at shutter closure. Progressively increasing resistance was added to the spirometer mouthpiece to simulate upper airway obstruction. The lung mechanics of 17 healthy subjects were successfully measured through spirometry.

**Results:**

*N* = 17 (8 female, 9 male) healthy subjects were recruited. Measured decay rates ranged from 5 to 42/s, subjects with large variation of decay rates showed higher muscular breathing effort. Lung elastance measurements ranged from 3.9 to 21.2 cmH$_2$O/L, with no clear trend between change in elastance and added resistance. Resistance calculated from decay rate and elastance ranged from 0.15 to 1.95 cmH$_2$Os/L. These very small resistance values are due to the airflow measured originating from low-resistance areas in the centre of airways. Occlusion resistance measurements were as expected for healthy subjects, and increased as expected as resistance was added.

**Conclusions:**

This test was able to identify reasonable dynamic lung elastance and occlusion resistance values, providing new insight into expiratory breathing effort. Clinically, this lung function test could impact current practice. It does not require high levels of cooperation from the subject, allowing a wider cohort of patients to be assessed more easily. Additionally, this test can be simply implemented in a small standalone device, or with standard lung function testing equipment.

## Background

Spirometry is the most frequently performed lung function test. It is a simple and low-cost test which assesses lung health by analysing airflow and lung volume during specific breathing manoeuvres [1–3]. The results of spirometry are able to guide therapy, by indicating the type and severity of any lung condition present.

However, underlying lung mechanics cannot be directly measured without further testing [4]. These mechanics are affected by lung disease, and monitoring how they change over time may provide a more accurate assessment of lung condition in response to therapy. Hence, there is a need to link easily obtained spirometry data with clinically and physiologically relevant lung mechanics models.

This proof-of-concept study presents a novel, model-based method of measuring lung mechanics during spirometry, or eventually with a standalone device. The model used is a dynamic elastance single-compartment lung model. The lung mechanics calculated are a constant, total system resistance, and a time-varying elastance. The dynamic elastance measured during expiration represents a combination of the lung’s elastic recoil and muscular expiratory effort. Lung mechanics were calculated by occluding expiratory breaths using a plethysmograph with built-in shutter, so occlusion resistance was also calculated.

Spirometry focuses on the results of the forced expiration manoeuvre. Patients are asked to inhale as deeply as possible, then exhale as forcefully as possible until forced vital capacity (FVC) is reached. This maximum effort breath effort can be difficult to achieve for small children and other patients with limited levels of cooperation or lung function. Additionally, large muscular expiratory effort can increase airway resistance as airways are constricted, and even cause gas trapping if small airways collapse.

Hence, the test described in this paper assesses the lung mechanics of tidal breathing. These mechanics represent lung condition at average breathing effort. Average expiratory effort may prove a better marker of lung condition, and be more readily measurable on a regular basis, than peak expiratory effort.

## Results

### Response to shuttering

**Fig. 1:**
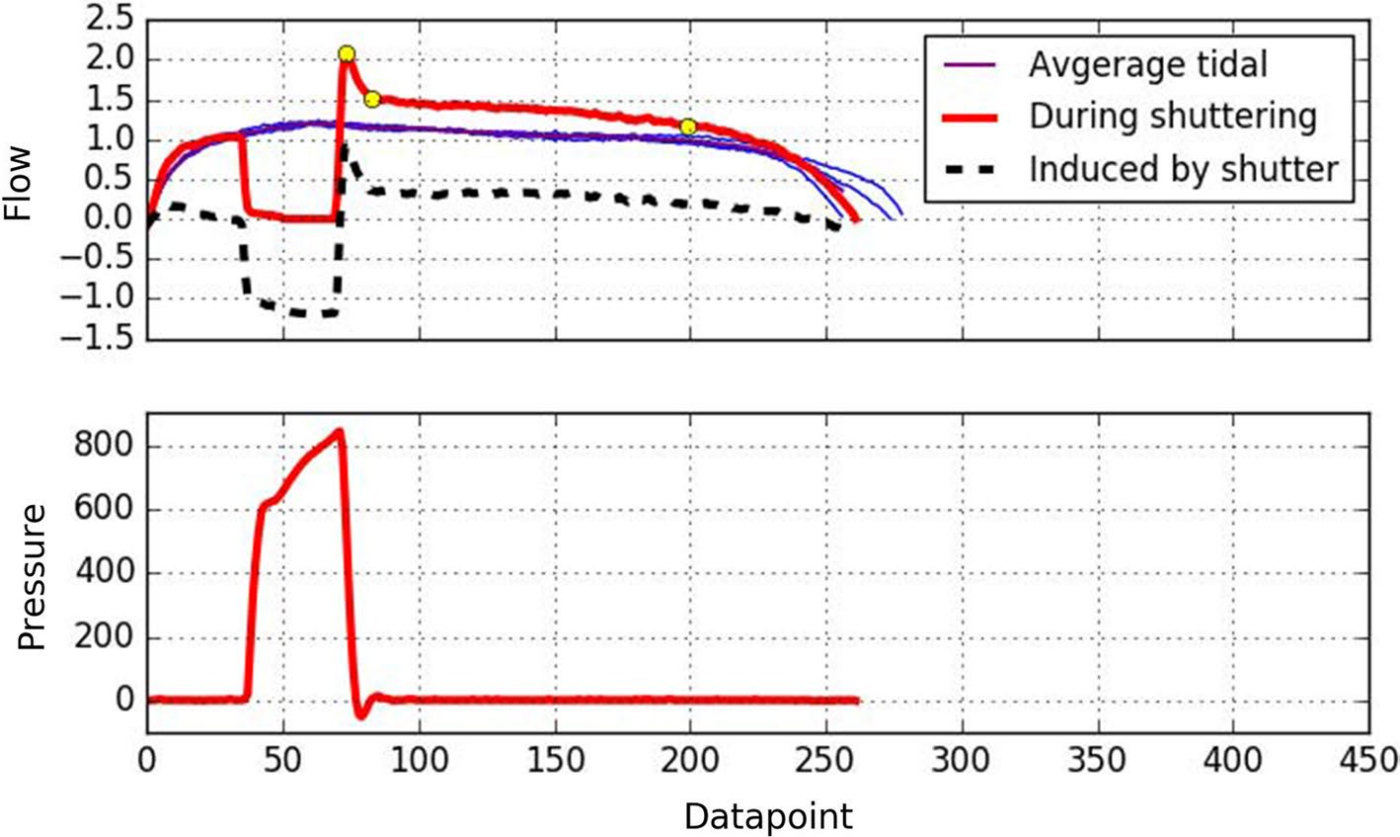
Flow and pressure traces measured during shuttering. Top: average tidal flow is shown in purple, measured flow is red, and the difference representing flow caused by shuttering is the dotted line. Bottom: pressure increases to approximate driving pressure while shutter is closed

Airflow measured during shuttering match simulated waveforms shown in Fig. 11. Figure 1 shows typical pressure and flow waveforms measured at baseline added resistance for comparison.

**Fig. 2:**
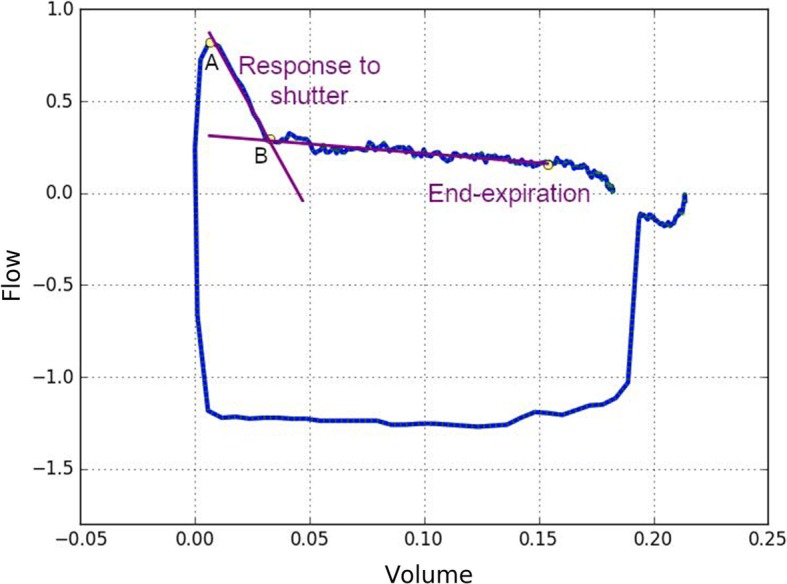
QV loop of flow induced by shutter makes identification of shuttering easy. A linear region between points A and B defined by the lung’s response to shutter reopening, and a longer region at end-expiration are shown. Note: linearity in a QV loop suggests lung mechanics remain constant. The total volume induced by the shutter has been translated in this figure to have a minimum value of zero

The average tidal flow is shown by the purple line in Fig. 1. This average flow was used to calculate flow caused by the shutter, which is shown by the dashed line in Fig. 1. The QV loop presented in Fig. 2 is for this airflow attributed to the shutter. After flow attributed to the shutter was expected to have decayed away, the shutter-induced flow was still greater than zero. This non-zero flow was due to the tidal flowrate remaining elevated above the expected average tidal flow rate for the entire duration after shuttering.

The response of a short linear region, typically 20–50 mL, followed by a long typically linear region with much lower or zero slope can be observed for all subjects at baseline added resistance. Non-linear and non-zero regions in the QV loop represent time-varying mechanics not present in the average tidal breathing waveform.

### Decay rates

The decay rate of shutter-induced flow was calculated from the QV loop. The exponentially decaying range was defined as from peak flow within 100 ms after shutter re-opening, to the first flow inflection point. The peak and inflection points are shown as points A and B, respectively, in Fig. 2. A linear least-squares fit was made to this data range. When collating all shuttered breaths, outliers were defined as data greater than 1.5 standard deviations above the 75th or below the 25th percentile. In addition, the decay rate was not calculated if the decaying range identified contained less than 3 datapoints. No other methods were used to exclude data from analysis.

**Fig. 3:**
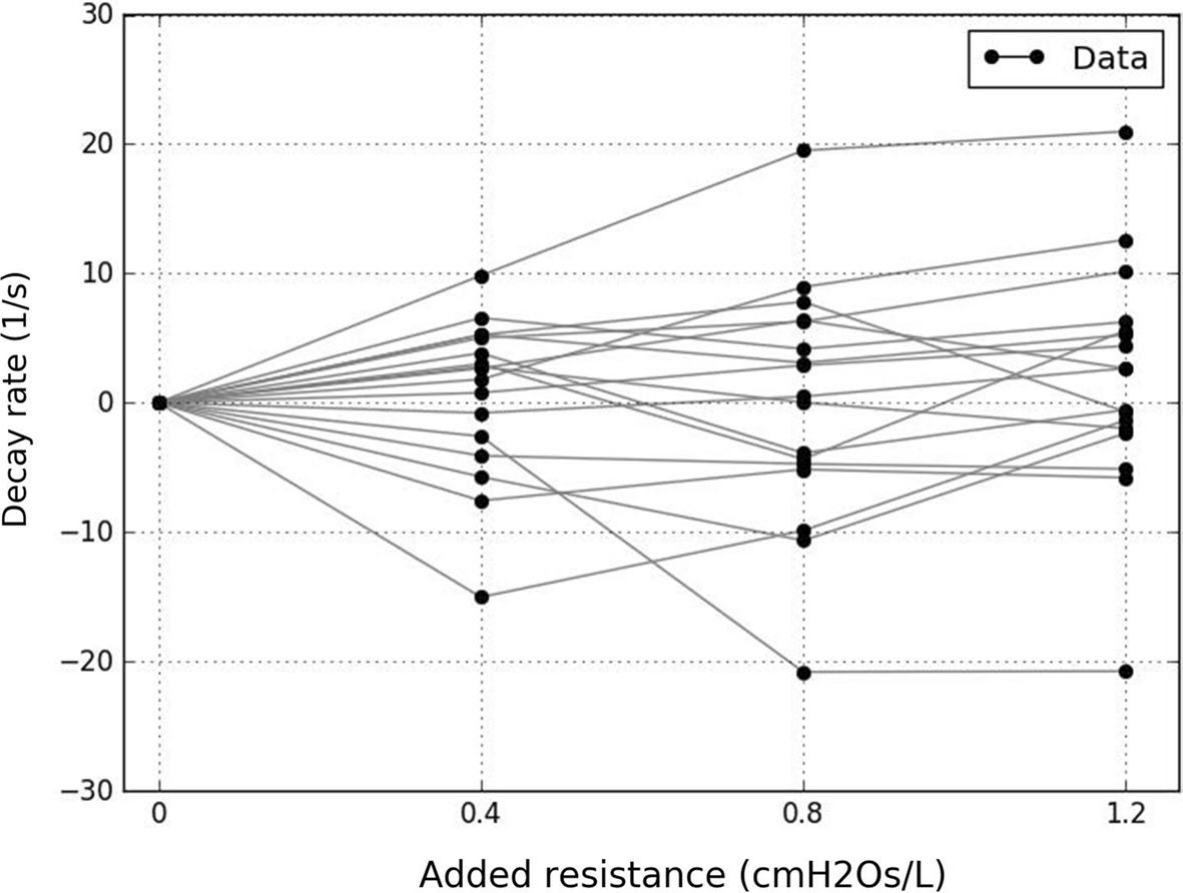
Decay rate trend for each subject. Decay rates are translated to 0/s at baseline. The decay rate did not consistently decrease as expected as resistance was added. Roughly half of the subjects saw increased decay rate at 1.2 cmH$_2$O added resistance, while the other half saw decreased decay rate

Table 1 contains details of the decay rates measured for all subjects for all external resistance levels. Decay rates measured range from 0.1 to 48. Flow decay rate is inversely proportional to resistance. So as resistance is added to the system, measured decay rate is expected to decrease. However, this trend was only observed for 7/17 subjects (Subjects 1, 3, 5, 11, 14, 16, 17), and only for the first three resistance levels (0, 0.4, 0.8 cmH$_2$Os/L). Figure 3 shows the trends observed for all subjects. No clear trend can be identified, with the average decay rate at each resistance level remaining unchanged.

**Table 1:** Mean decay rate and standard deviation measured for all subjects at all resistance levels

Subject	Decay rate (1/s, mean [std]) at added resistance (cmH$_2$Os/L)			
	None	0.4	0.8	1.2
1	− 15.52 [2.99]	− 12.75 [3.17]	− 15.50 [4.83]	− 17.51 [7.19]
2	− 33.17 [9.25]	− 48.21 [20.57]	− 43.05 [23.85]	− 34.53 [17.81]
3	− 33.75 [8.04]	− 23.92 [5.24]	− 14.25 [7.71]	− 12.77 [5.37]
4	− 22.13 [3.41]	− 22.93 [2.69]	− 21.66 [9.19]	− 19.48 [5.58]
5	− 14.43 [6.16]	− 9.18 [4.50]	− 11.35 [5.94]	− 9.19 [6.59]
6	− 19.30 [3.37]	− 16.30 [4.80]	− 23.66 [8.47]	− 13.72 [9.48]
7	− 24.70 [6.56]	− 20.86 [7.52]	− 28.59 [10.13]	− 25.26 [7.07]
8	− 13.92 [3.08]	− 21.50 [16.65]	− 19.08 [3.78]	− 19.72 [12.66]
9	− 0.13 [4.35]	− 2.74 [4.71]	− 20.95 [19.28]	− 20.89 [26.62]
10	− 16.86 [5.02]	− 22.60 [6.51]	− 27.53 [6.48]	− 19.23 [7.09]
11	− 26.33 [6.46]	− 21.32 [5.21]	− 20.06 [3.52]	− 16.18 [13.86]
12	− 7.85 [2.25]	− 11.96 [3.50]	− 12.59 [6.51]	− 12.98 [9.34]
13	− 15.64 [4.10]	− 9.09 [1.93]	− 11.49 [4.35]	− 9.41 [4.42]
14	− 30.66 [4.53]	− 28.86 [3.67]	− 21.72 [3.77]	− 18.06 [5.40]
15	− 30.58 [9.52]	− 25.34 [3.73]	− 22.78 [2.55]	− 31.29 [14.30]
16	− 17.94 [3.13]	− 17.17 [3.51]	− 15.05 [4.57]	− 13.56 [6.06]
17	− 21.11 [6.23]	− 18.46 [8.75]	− 14.74 [8.32]	− 18.45 [17.68]

Decay rates are defined as negative, due to driving pressure creating negative flow

**Fig. 4:**
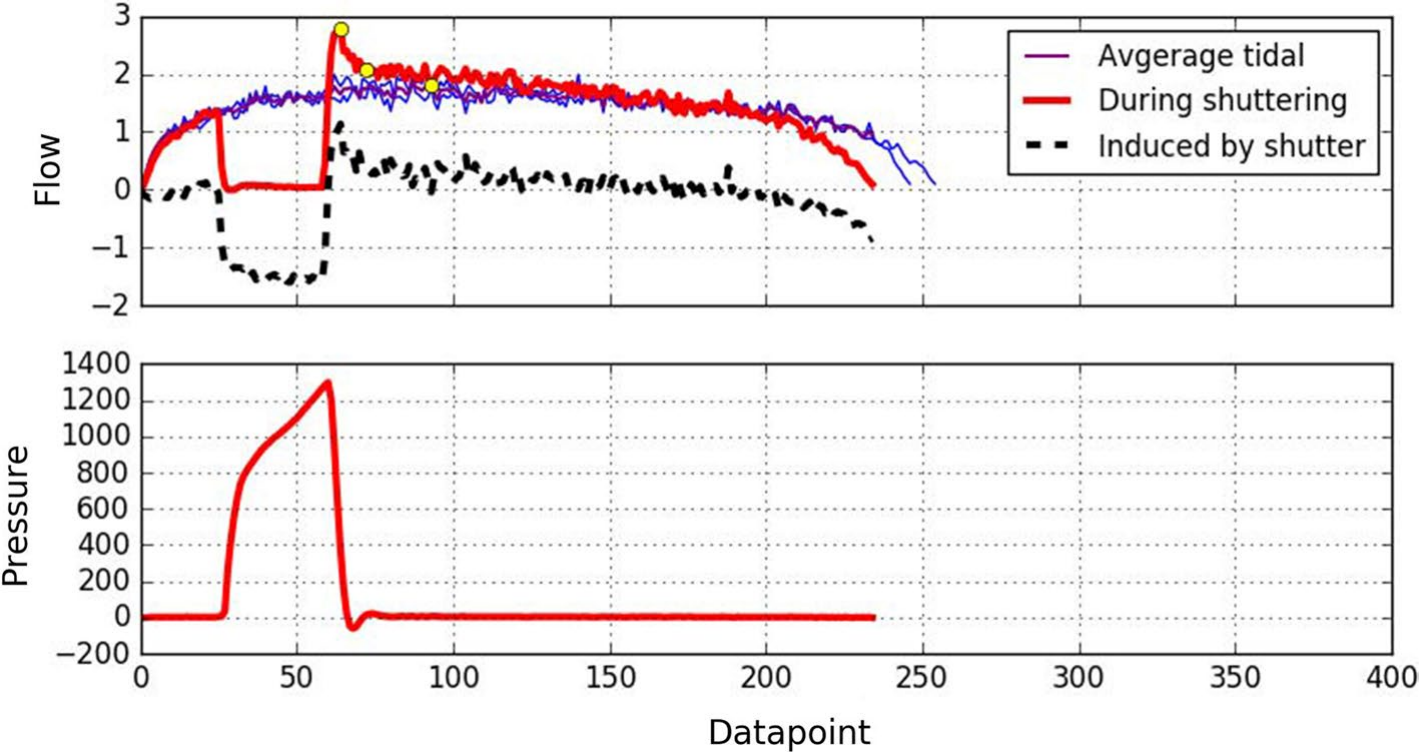
Large amounts of noise were added to the flow signal when a venturi was added in series before the flow sensor. Three venturis were used in this study, with constrictions of diameter diameters of 9.5 mm, 10.5 mm and 12.5 mm

Additional resistance was added to the spirometer mouthpiece via venturis specifically designed for this study. The venturi was inserted between the mouthpiece and flow sensor. This positioning added noise to the flow measurements due to turbulent airflow from the venturi. An example is shown in Fig. 4. This noise increased with increasing resistance, and was large enough for some subjects to obscure the underlying waveform, preventing any measurements of decay rate.

### Mechanics

**Fig. 5:**
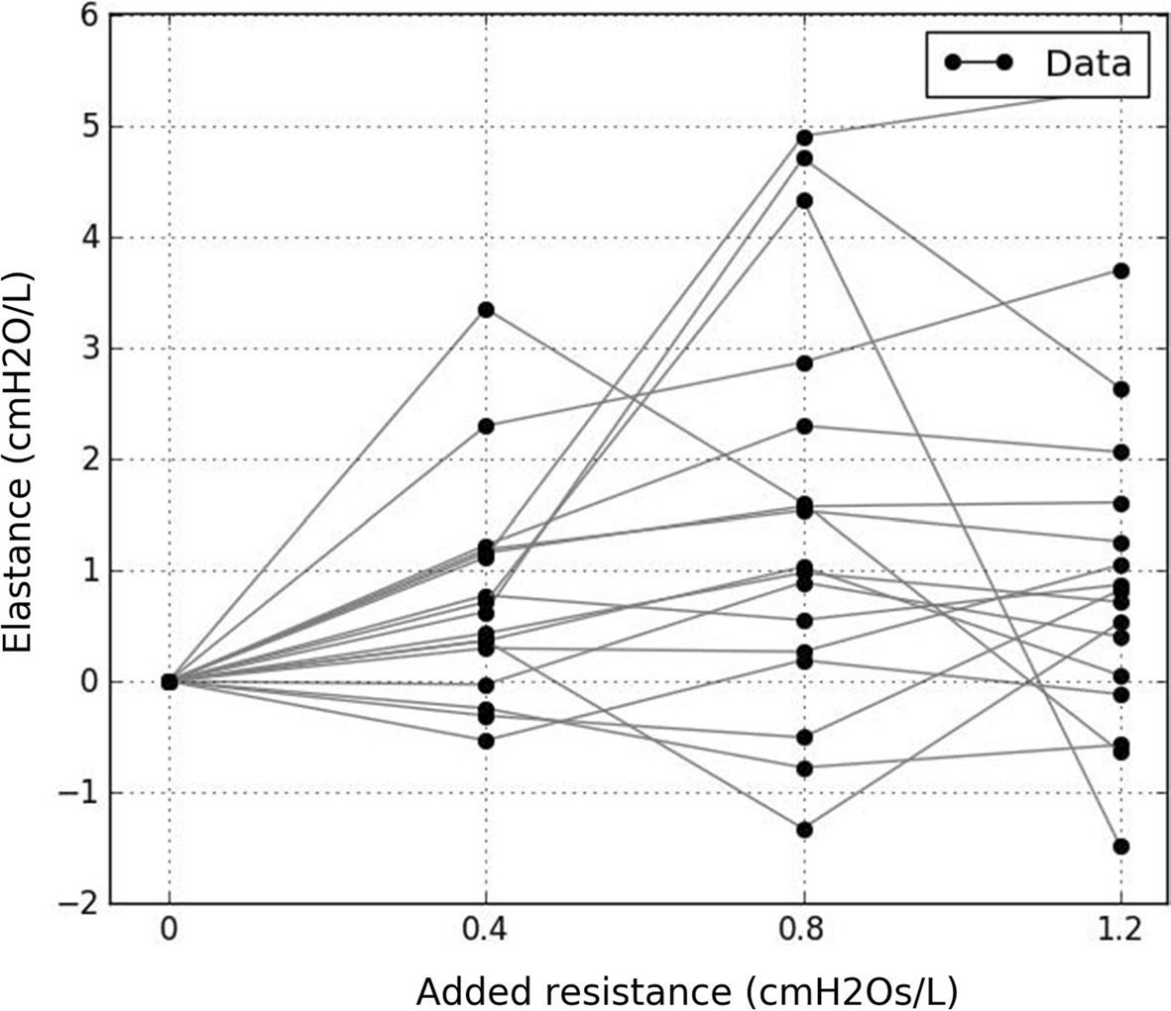
Elastance trend with added resistance observed for all subjects. Elastance is translated to 0  cmH$_2$O/L at baseline. No clear elastance trend is seen for increasing added resistance. However, elastance is likely to increase as external resistance increases

Lung elastance calculated at shutter opening is presented in Table 2, and the effect of added resistance on elastance is shown in Fig. 5. Elastance values range from 3.9 to 22.1 cmH$_2$O/L, and generally showed small intra-subject variation for each resistance level with a typical standard deviation less than 1.0 cmH$_2$Os/L. No clear trend was observed between elastance and added resistance, with elastance remaining fairly consistent between resistance levels.

**Table 2:** Dynamic elastance (mean [STD dev]) identified for each subject for each resistance level

Subject	Ed (mean [std]) cmH$_2$O/L			
	None	0.4	0.8	1.2
1	5.36 [0.45]	6.14 [0.47]	5.91 [0.14]	6.23 [0.42]
2	4.78 [0.73]	4.47 [0.48]	4.27 [0.39]	5.60 [0.56]
3	5.98 [0.44]	6.34 [0.30]	7.01 [1.74]	6.03 [0.63]
4	7.62 [0.61]	8.06 [0.60]	8.60 [0.77]	8.34 [0.46]
5	7.68 [1.14]	9.98 [1.43]	10.56 [1.18]	11.39 [1.73]
6	6.41 [0.60]	6.16 [0.27]	5.63 [0.19]	5.83 [0.36]
7	3.93 [0.35]	4.23 [0.54]	4.20 [0.45]	4.98 [0.54]
8	5.75 [0.86]	5.22 [1.01]	5.94 [0.65]	5.63 [0.86]
9	7.62 [2.54]	8.25 [1.89]	12.34 [2.81]	10.26 [1.43]
10	8.27 [1.20]	8.64 [0.64]	6.95 [1.18]	8.81 [1.04]
11	14.84 [3.94]	18.19 [3.10]	16.44 [2.97]	14.20 [1.15]
12	8.92 [1.32]	10.15 [1.32]	11.22 [1.77]	10.99 [0.74]
13	17.73 [1.35]	18.44 [2.75]	22.07 [2.77]	16.24 [3.51]
14	14.53 [1.18]	15.72 [0.61]	16.07 [0.87]	15.79 [1.14]
15	7.76 [0.97]	8.88 [1.22]	12.68 [2.50]	13.10 [0.80]
16	17.44 [2.12]	17.42 [1.78]	18.34 [2.95]	17.85 [3.10]
17	8.94 [0.94]	10.10 [1.50]	10.52 [1.17]	10.55 [1.09]

Elastance was measured at shutter re-opening

**Fig. 6:**
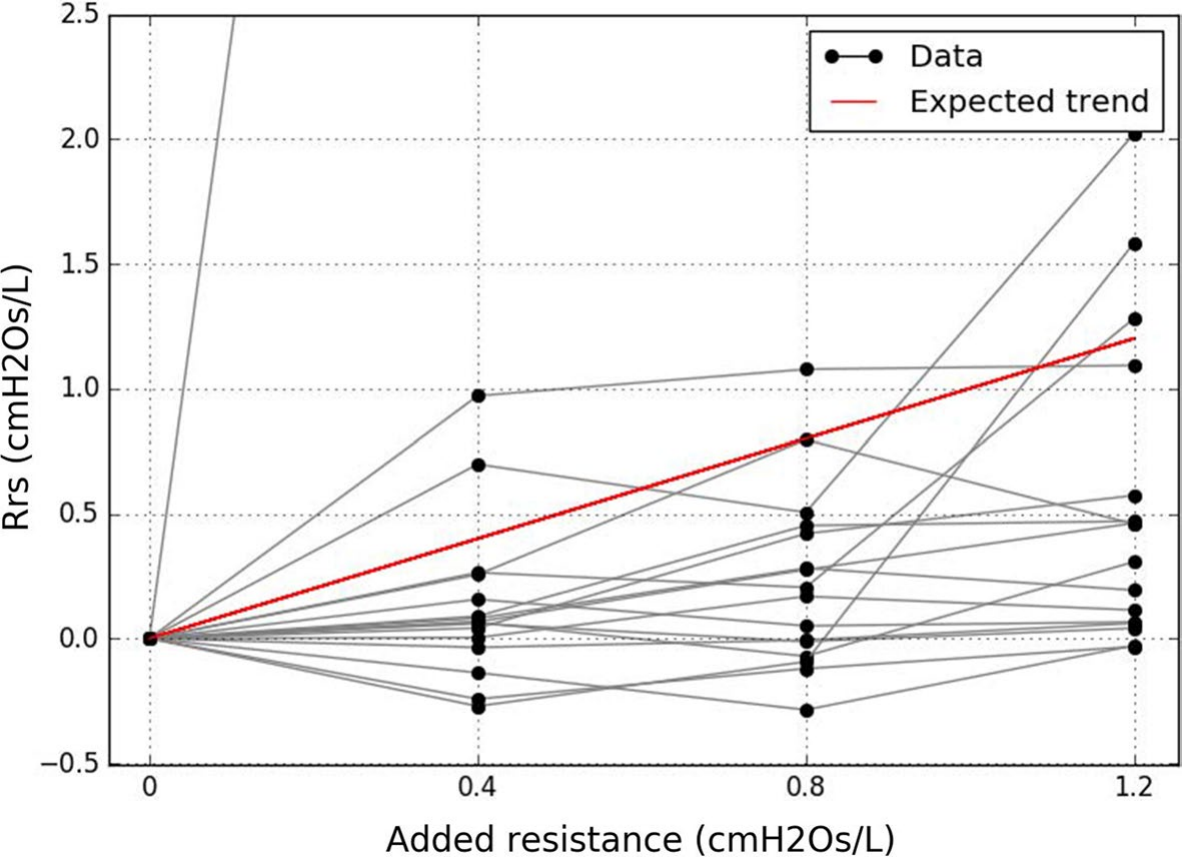
$R_{\text {rs}}$ trend with added resistance observed for all subjects. $R_{\text {rs}}$ is translated to 0  cmH$_2$Os/L at baseline. No clear trend is seen for increasing added resistance. $R_{\text {rs}}$ measured at each added resistance level was expected to rise by the same amount added

$R_{\text {rs}}$ separated from flow decay rate is presented in Table 3, and Fig. 6 shows how $R_{\text {rs}}$ changes with added resistance. At all levels of added resistance, excluding Subject 9, measured $R_{\text {rs}}$ was very small. The largest resistance of 2.84 cmH$_2$Os/L was measured for Subject 12. Generally, $R_{\text {rs}}$ was less than the value of added resistance alone. $R_{\text {rs}}$ does not increase proportional to added resistance, as had been expected.

**Table 3:** Resistance (mean [STD dev]) identified from decay rate at each added resistance level

Subject	$R_{\text {rs}}$ (mean [std]) cmH$_2$Os/L			
	None	0.4	0.8	1.2
1	0.36 [0.10]	0.52 [0.17]	0.42 [0.11]	0.43 [0.20]
2	0.16 [0.06]	0.12 [0.08]	0.15 [0.12]	0.22 [0.12]
3	0.19 [0.04]	0.28 [0.05]	0.64 [0.32]	0.66 [0.47]
4	0.35 [0.06]	0.36 [0.05]	0.52 [0.34]	0.47 [0.14]
5	0.70 [0.47]	1.40 [0.72]	1.20 [0.61]	2.72 [2.97]
6	0.34 [0.08]	0.41 [0.11]	0.27 [0.10]	0.65 [0.37]
7	0.18 [0.07]	0.24 [0.10]	0.16 [0.05]	0.22 [0.08]
8	0.45 [0.16]	0.20 [0.45]	0.33 [0.08]	0.41 [0.24]
9	− 12.45 [32.65]	− 2.81 [10.44]	1.48 [1.20]	− 1.19 [2.82]
10	0.55 [0.23]	0.42 [0.12]	0.27 [0.07]	0.52 [0.20]
11	0.64 [0.32]	0.90 [0.26]	0.84 [0.21]	1.92 [1.61]
12	1.25 [0.43]	0.98 [0.50]	1.16 [0.60]	2.84 [4.54]
13	1.22 [0.37]	2.19 [0.86]	2.30 [1.08]	2.31 [1.52]
14	0.49 [0.09]	0.55 [0.06]	0.76 [0.13]	0.95 [0.26]
15	0.28 [0.09]	0.36 [0.08]	0.56 [0.12]	0.47 [0.12]
16	1.01 [0.25]	1.05 [0.20]	1.43 [0.73]	1.58 [0.76]
17	0.47 [0.16]	0.72 [0.41]	1.26 [1.12]	0.93 [0.45]

Elastance used to separate decay rate is shown in Table 2

Expiratory elastance calculated by this test is presented as positive, due to the spirometer defining positive pressure as expiration. When pressure is typically measured for calculating lung mechanics, such as during mechanical ventilation, positive pressure is defined as inspiration. As such, the negative of elastance was used to separate resistance from decay rate.

**Fig. 7:**
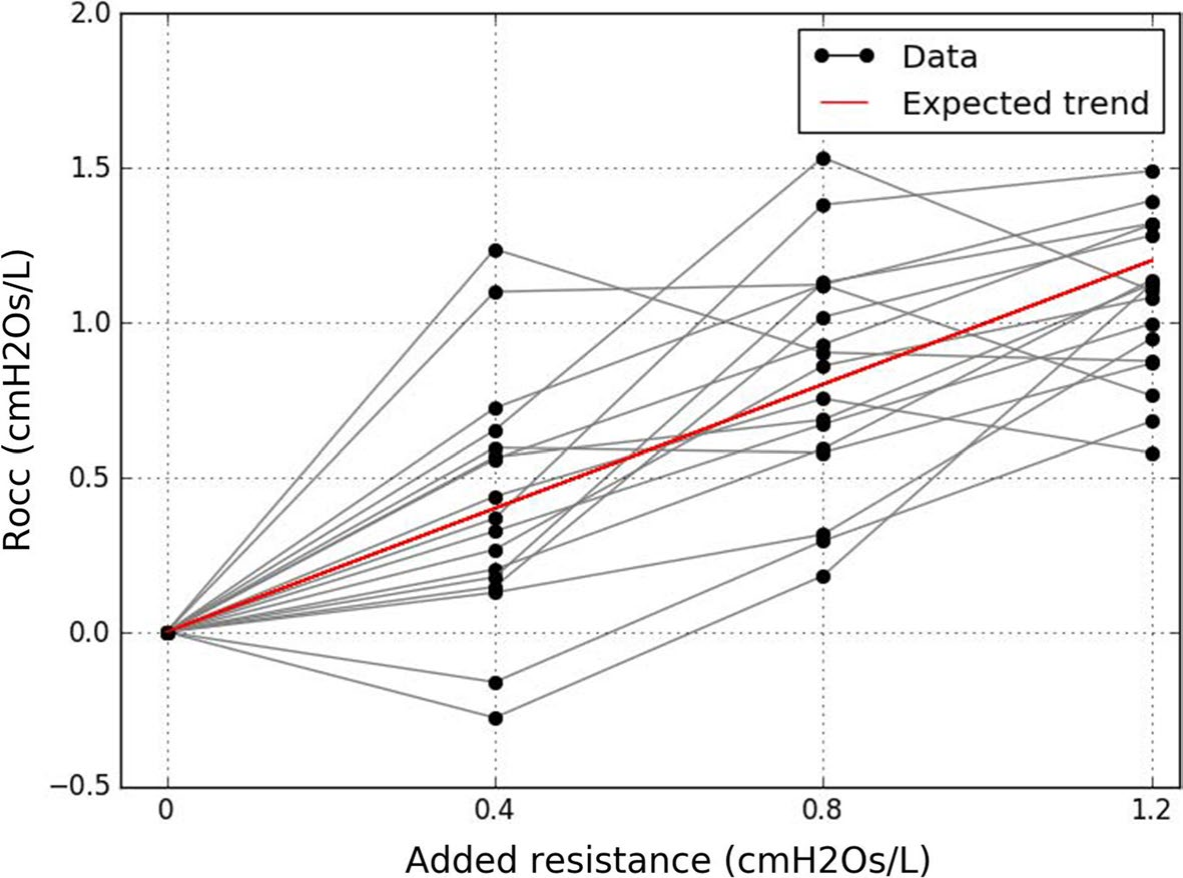
ROCC trend with added resistance observed for all subjects. ROCC is translated to 0  cmH$_2$Os/L at baseline. Generally, ROCC measured at each added resistance level generally increased by the same amount added

Table 4 shows ROCC calculated for each subject, and the effect of external resistance on ROCC is shown in Fig. 7. ROCC ranged from 3.0 to 8.0. Measured resistance is expected to increase proportional to the added resistance. On average, ROCC results match this expectation. However, the deviation from expected ROCC could be quite large, at ±1 cmH$_2$Os/L for this study.

**Table 4:** Occlusion resistance was calculated for each subject at each resistance level

Subject	Rocc (mean [std]) cmH$_2$Os/L			
	None	0.4	0.8	1.2
1	3.33 [0.55]	3.05 [0.19]	3.51 [0.28]	4.45 [0.79]
2	3.45 [0.36]	3.60 [1.12]	4.47 [1.30]	4.73 [0.95]
3	3.38 [0.25]	3.94 [0.34]	4.31 [0.46]	4.70 [0.86]
4	3.70 [0.73]	3.82 [0.38]	4.01 [0.34]	4.64 [1.15]
5	4.56 [0.62]	4.89 [0.59]	5.23 [0.56]	5.56 [0.65]
6	5.83 [0.60]	6.42 [0.32]	6.41 [0.57]	6.70 [0.49]
7	3.41 [0.31]	3.97 [0.34]	4.09 [0.32]	4.53 [0.55]
8	5.37 [0.88]	5.21 [0.55]	5.66 [0.78]	6.05 [0.70]
9	3.64 [0.29]	3.84 [0.36]	4.23 [0.47]	4.78 [0.89]
10	3.58 [0.27]	4.02 [1.33]	4.34 [0.41]	4.16 [0.47]
11	4.54 [0.38]	4.80 [0.24]	5.40 [0.46]	5.62 [0.75]
12	3.97 [0.29]	5.20 [1.88]	4.87 [0.47]	4.84 [0.45]
13	6.47 [0.39]	6.84 [1.16]	7.85 [1.09]	7.96 [0.92]
14	4.17 [0.19]	5.27 [1.70]	5.29 [1.15]	4.93 [0.61]
15	3.58 [0.30]	3.75 [0.34]	4.71 [0.72]	4.90 [0.53]
16	4.32 [0.35]	5.05 [0.51]	5.45 [0.57]	5.72 [0.77]
17	4.43 [0.51]	5.08 [0.47]	5.96 [0.66]	5.53 [0.80]

ROCC is expected to increase by 0.4 cmH$_2$Os/L per external resistance level

## Discussion

### Decay rates

The exponentially decaying region was easily located for all subjects, except Subject 9. The shutter duration used in this study (200 ms) was twice as long as the minimum required. Longer shutter duration gives more time for subjects to react to the shutter. Subject 9 appeared to react to shuttering by significantly reducing respiratory effort after approximately 100 ms of shutter closure, or an air-leak was created around the mouthpiece. This flow reduction resulted in incorrect measurements of decay rate, and, consequently, for the resistance calculated from the decay rate.

**Fig. 8:**
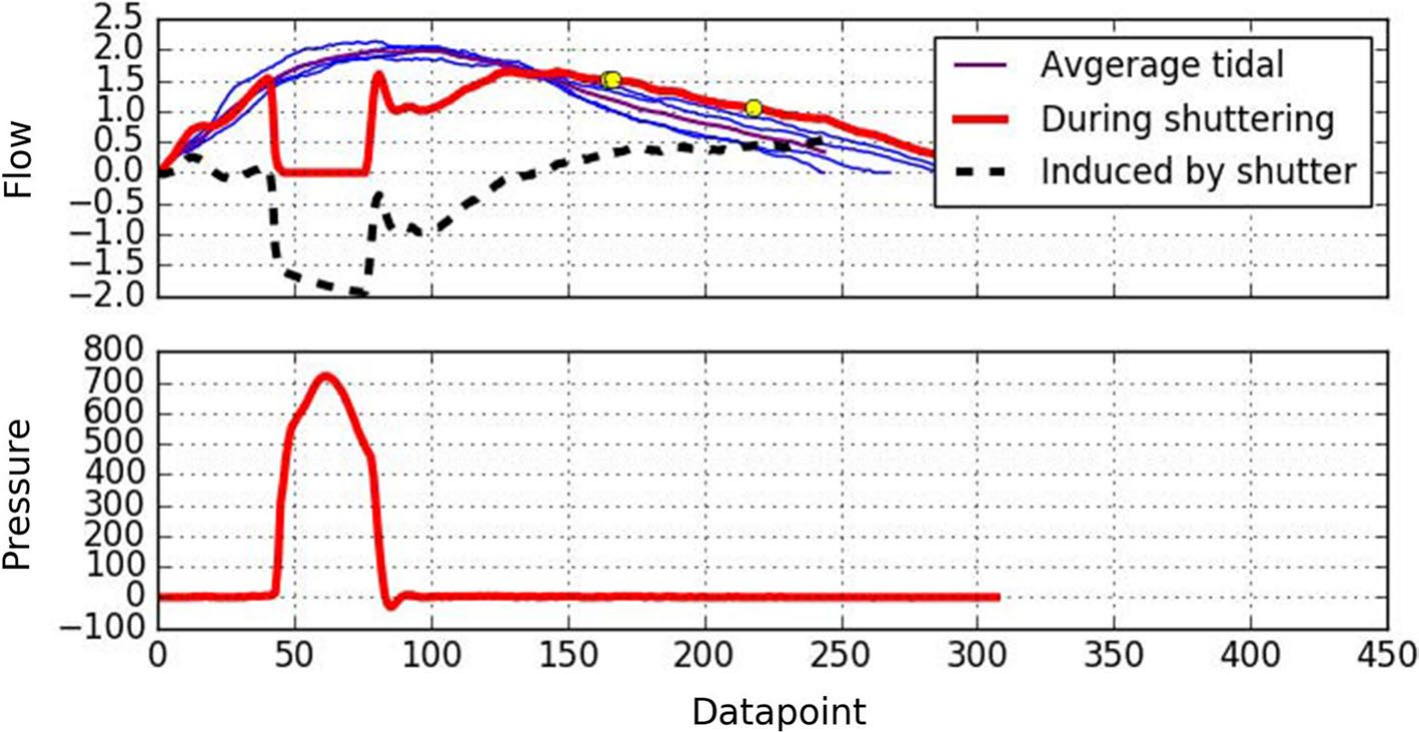
Flow measurements for Subject 9 were lower than expected after shutter release. Airflow measured after shuttering was less than average tidal airflow (purple line) and pressure reduced during shutter closure, indicating a muscular reflex in response to the shutter

An assumption used in data analysis was the airflow in response to shuttering would be superimposed on average tidal breathing. Due to the reduction in airflow for Subject 9, the effective airflow induced by the shutter is negative, as seen in Fig. 8. As a result, the correct region could not be identified.

In general, the measured decay rates had fairly large intra-subject variation with the standard deviation often as high as 30% of the mean value. Subjects 2 and 3 in particular had extremely large variation in measured decay rate. These subjects had a variety of different looking post-shutter waveforms, but no significant reduction in driving pressure. However, the changes in airflow shape indicate possible muscular reaction to shuttering. Hence, even though the decaying flow is correctly identified, the decay rate calculated may be incorrect, because it is not possible to separate the effects of the shutter from unexpected muscular reaction.

The degree of muscular reaction to shuttering is expected to decrease with shorter occlusion duration. Hence, higher breath-to-breath consistency should be achieved by decreasing the duration closer to 100 ms. In addition, subjects in this study were required to consciously breathe deeper than usual due to minimum flow rate limitations. If this limit were reduced or removed, breath-to-breath variation may reduce, because each breath could be driven subconsciously.

### Mechanics

The expected range of static elastance for healthy lungs is 2–10 cmH$_2$Os/L [5–7]. Elastance measured in this study generally fit into this expected range, indicating the majority of airflow could be attributed to lung elastic recoil. There was no consistent trend between measured elastance and added resistance. However, elastance tended to be slightly higher when external resistance was added. These results suggest external resistance and the use of shuttering may not significantly affect measured elastance, but some breath-to-breath elastance change should be expected.

Elastance measured for Subjects  11, 13, 14, and 16 was higher than expected. For healthy, tidally breathing subjects, increased elastance indicates muscular breathing effort. The additional elastance due to muscular effort cannot be separated from static elastance without further measures. Hence, expiratory breathing effort during mechanics measurement could indicate restrictive disease where none is present. However, the measurement of combined elastance of tidal breathing may provide clinically relevant information. A larger than expected elastance during quiet tidal breathing indicates an elevated work-of-breathing, which could negatively impact quality of life.

$R_{\text {rs}}$ was much smaller than expected. The typical range of airway resistance for healthy subjects is around 1.5–2.5 cmH$_2$Os/L [8, 9]. However, $R_{\text {rs}}$ was less than 1.3 cmH$_2$Os/L for all subjects at baseline added resistance. Often, $R_{\text {rs}}$ calculated was less than added external resistance alone. $R_{\text {rs}}$ was not significantly or consistently affected by added external resistance. Airflow begins in areas in the centre of airways with low skin friction effects. Because airflow induced by the shutter decays quickly, typically only 20–50 ml of air is involved. The small volume does not allow enough time for air to flow from higher friction areas, resulting in a poor airway resistance estimate. As a result, monitoring decay rate of flow in response to shuttering or other pressure impulses only gives information on lung elastance. However, separate resistance measurements are possible during shuttering.

Rocc is an established method to measure airway resistance during tidal breathing. The combined resistance of plethysmograph and mouthpiece was 1.5 cmH$_2$Os/L. Subtracting this resistance from Rocc measured at 0 cmH$_2$Os/L added resistance, all subjects fell into the range 1.9–5.2 cmH$_2$Os/L. Rocc increased as external resistance was added. However, the increase in Rocc was not consistently the 0.4 cmH$_2$Os/L expected, showing a limitation of the single-compartment model.

### Extrapolating mechanics

**Fig. 9:**
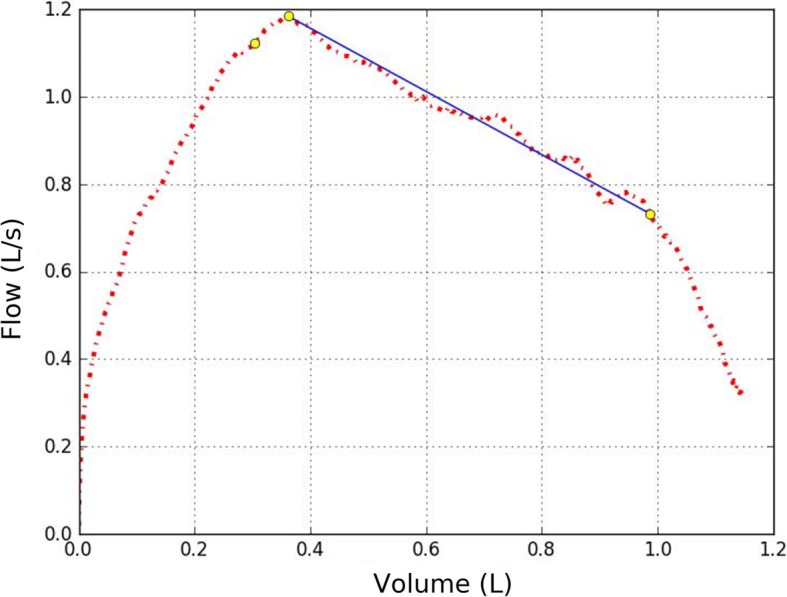
Expiration is not an entirely passive process, as seen by non-linearity between flow and volume. This QV loop is the top half of the typically analysed spirometry QV loop, as only expiration is assessed. However, the end-expiratory portion shows a generally linear trend, indicating lung mechanics do not vary in this portion

In general, the average tidal QV loop for all subjects showed a linear end-expiratory relationship between flow and volume, as shown in Fig. 9. This result suggests healthy lung mechanics of end-expiration are relatively constant during tidal breathing. Hence, when instantaneous mechanics are measured during end-expiration, as in this study, the mechanics can be expected to represent the entire end expiratory portion.

### Limitations

Without further measures, the contribution of static lung tissue elastance cannot be separated from the dynamic elastance created by muscular breathing effort. Subjects in this study showed a range of breathing effort. Increased breathing effort was shown to clearly obscure passive elastance in some cases. However, subjects with respiratory illness, such as COPD, may exhibit less end-expiratory breathing effort, as their capacity for breathing effort is reduced.

The shutter closure duration was quite long, at 200–250 ms. Reducing occlusion to 100 ms may lead to more consistent intra- and inter-subject results. In particular, because there is less time for subjects to react to shuttering, the adaption to shuttering would be limited. Hence, there is a tradeoff between shutter duration, sampling rate, and sensor noise to assess mechanics in this way.

Although QV loops of tidal breathing suggest lung mechanics remain fairly constant at end-expiration, the shutter may cause changes in lung mechanics. The large pressure built-up in the respiratory system during occlusion may cause airways to increase in diameter, reducing their resistance to airflow. Additionally, lung viscoelasticity tends to increase the pressure during shutter closure. The viscoelastic pressure build-up may increase measured elastance calculated at shutter re-opening.

Access to a plethysmograph, as used in this study, or a spirometer with built in shutter may be limited. To broaden access to this test, a simple, portable, hand-held device could be used to measure these lung mechanics.

## Conclusions

This proof-of-concept study describes a novel method to non-invasively measure lung mechanics of tidally breathing subjects. This test was able to identify reasonable dynamic lung elastance along with Rocc. Measurements of both mechanics were within the expected physiological range for healthy subjects breathing with elevated muscular effort. Rocc was able to follow changes in upper airway resistance with some subject-dependent variation, as expected. Clinically, this lung function test could impact current practice. It does not require high levels of cooperation from the subject, allowing a wider cohort of patients to be assessed. Additionally, this test could be widely accessible as it can be implemented with either a small standalone device, or standard lung function testing equipment.

## Methods

### Linear single-compartment lung model

The single-compartment lung model is simple and easy to understand [10]. This model has been further developed to give the dynamic elastance, single-compartment lung model, which describes the elastic properties of the lung as a combination of static and time-varying components [11]. This model has been used in the intensive care unit (ICU) to estimate lung mechanics for mechanically ventilated patients spontaneously breathing on top of ventilator support [11, 12]. The model has also been extended to predict future lung condition for adult ICU patients [13, 14]

The model used in this study is the dynamic elastance, single-compartment lung model. Respiratory muscles produce the driving pressure needed to oppose passive respiratory elastic forces, such as lung and chest recoil, and maintain airflow through respiratory airways. Hence, the model is defined:

1 $$\begin{aligned} E_{\text {dy}}(t)V(t) = E_{\text {rs}}V(t) + R_{\text {rs}}Q(t), \end{aligned}$$

where $E_{\text {dy}}$ is the dynamic elastance representing muscular breathing effort to create driving pressure, $E_{\text {rs}}$ is the sum of all passive respiratory system elastances, $R_{\text {rs}}$ is the combination of respiratory airway resistance and external resistances, *V* is volume, *Q* is flow and *t* is time,

Direct measurement of respiratory driving pressure, $E_{\text {dy}}(t)V(t)$, is not possible without highly invasive measures, such as an oesophageal balloon catheter. Without direct measurement of driving pressure, lung mechanics cannot be simply identified. As an example, variations in individual lung mechanics can cause identical airflow in different subjects to be be created by vastly different driving pressures.

This study makes use of two properties of the lung predicted by the single-compartment lung model. First, an exponentially decaying flow will be created in response to a large, sudden change in driving pressure [15]. This property can be shown by combining $E_{\text {dy}}$ and $E_{\text {rs}}$ into a total driving elastance term, $E_{\text {d}}$. The result is a simple lung model describing the balance of elastic and resistive force in the lung:

2 $$\begin{aligned} E_{\text {d}}(t)V(t) = R_{\text {rs}}Q(t). \end{aligned}$$

Solving the resulting ODE for *Q*(*t*) yields:

3 $$\begin{aligned} Q(t) = Q_0\text {e}^{\frac{-tE_{\text {d}}(t)}{R_{\text {rs}}}}. \end{aligned}$$

Equation 3 shows the decay rate of airflow in response to a change in driving pressure depends on a combination of the lung mechanics terms, $E_{\text {d}}$ and $R_{\text {rs}}$.

A second property predicted by the single-compartment lung model is that when there is no airflow, any pressure measured at the mouth must be due to elastic lung mechanics. If the airways are held open to the atmosphere, lung recoil and muscular effort will balance at atmospheric pressure. However, if breathing is occluded, the pressure measured at the mouth will be equal to the combination of driving pressure and lung recoil.

### Mechanics identification

A shutter built into a plethysmograph was used to induce exponentially decaying flow. The shutter occluded expiration for 200 ms, which is longer than the 100 ms minimum needed for pressure to equalise across the respiratory system [16]. When the shutter was released, pressure at the mouth dropped from driving pressure to atmospheric pressure, creating the exponentially decaying flow described by Eq. 3.

**Fig. 10:**
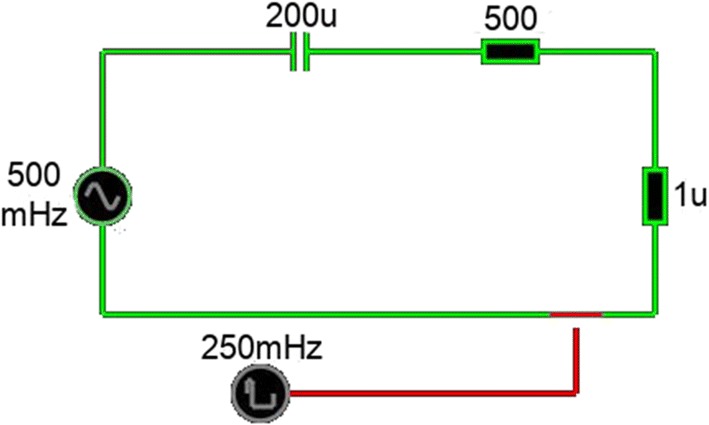
Electrical model of respiratory system. Shutter at mouth is modelled with with voltage-controlled switch. Component values were chosen to approximately match human respiratory mechanics (*C* = 200 $\mu$F, Raw = 500 Ohm, frequency = 0.5 Hz, shutter duration = 200 ms)

**Fig. 11:**
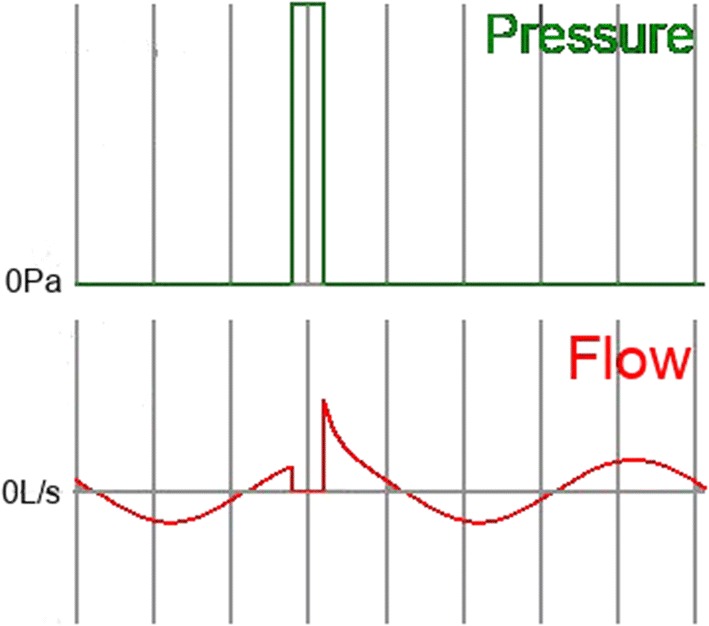
Response of electrical lung model. Top: signal to simulate shuttering. Bottom: modelled flow in spirometer. The response to shutter re-opening is the superposition of sinusoidal flow due to respiratory muscles, and an exponential decay induced by the large change in pressure

The lung’s response to shuttering can be simulated with an electrical circuit, with the shutter modelled as a voltage-controlled switch, as shown in Fig. 10. Figure 11 shows the simulated pressure and airflow measurements. The airflow measured after the shutter is re-opened is a superposition of tidal flow due to respiratory muscles and exponentially decaying flow caused by the shutter.

The exponentially decaying airflow can be separated from the measured flow using adaptive filtering. The breath-to-breath variation of tidal breathing is low, with breaths typically having similar magnitude, duration, and flow profile. Hence, the expected tidal airflow can be estimated by averaging all breaths before the shuttered breath. In this study, a minimum of 5 tidal breaths were recorded before each shuttered breath. The airflow created in response to the shutter re-opening is calculated by subtracting the average tidal airflow from airflow measured during shuttering.

An assumption of the single-compartment lung model is that passive lung mechanics do not change during the breath. If muscular breathing effort also remains constant after the shutter is re-opened, the decay rate of flow caused by the shutter, $E_{\text {d}}/R_{\text {rs}}$, can be calculated with a linear fit to the trace of airflow vs volume (QV loop). However, the elastance and resistance cannot be separated by measuring the decay rate alone. An additional measure of either $R_{\text {rs}}$ or $E_{\text {d}}$ is required to separate the combined mechanics of the decay rate  [17–19].

Elastance is defined as the pressure in the lung per unit volume. Pressure measured while airflow is occluded will approximate the respiratory driving pressure. Hence, if muscular effort remains constant, the elastance after the shutter re-opens can be calculated from the pressure and volume measured momentarily before the shutter re-opens ($P_0$ and $V_0$, respectively):

4 $$\begin{aligned} P_0 = E_\text {d}(t) V_0. \end{aligned}$$

$R_{\text {rs}}$ was able to be separated from the decay rate after this elastance was calculated.

Additionally, occlusion resistance (ROCC) was calculated following standard protocol [16, 20]. The gradient of pressure from 30 to 75 ms after the shutter was closed was extrapolated backwards to 15 ms before closure. The difference between this extrapolated pressure and the true pressure measurement at that time was divided by the airflow recorded at that time to produce an estimate for total airway resistance before occlusion.

Due to minimum flow limits built into the shuttering software employed, lung mechanics were measured while panting. For each test, the shutter was activated 5 times with a minimum of 5 normal breaths recorded before shuttering. Extra resistance was added to the spirometer mouthpiece to simulate upper airway obstruction. The test was repeated twice at each resistance level. The resistances added were 0 (baseline), 0.4, 0.8, and 1.2 cmH$_2$Os/L, respectively.

### Data

Seventeen healthy subjects were enrolled in this study (8 female, 9 male, age 27±4.5, BMI 25±4, 3 smokers), where subjects were deemed healthy if they had no current respiratory disease or history of severe respiratory disease. Smokers were included in this study. Data were recorded using a Ganshorn PowerCube Body plethysmograph with LFX 1.8 Respiratory Diagnostic Software. The Shutter was controlled with LFX software’s ROCC mode, in manual trigger mode with a shutter close duration of 200–250 ms (typically 200 ms). Table 5 shows specific details for each subject.

**Table 5:** Subject data

Subject	Sex	Age	Height (cm)	Weight (kg)	Smoker
1	M	30	190	100	n
2	M	38	175	100	n
3	M	32	187	87	n
4	M	29	183	95	n
5	F	24	173	80	y
6	M	29	183	78	n
7	M	23	185	73	y
8	M	23	184	71	n
9	M	27	178	90	n
10	F	29	168	62	n
11	F	22	167	53	n
12	F	29	161	53	y
13	F	23	164	64	n
14	F	25	172	70	n
15	M	31	181	114	n
16	F	21	164	72	n
17	F	21	160	56	n

Smokers were included in this study

## Data Availability

The datasets generated and analysed during the current study are not publicly available for privacy reasons but anonymised data are available from the corresponding author on reasonable request.

## Abbreviations

ICU: Intensive care unit
Rocc: Occlusion resistance
COPD: Chronic obstructive pulmonary disease
$R_{\text {rs}}$: Respiratory system resistance
ROCC: Occlusion resistance

## References

[1] Coates AL, Tamari IE, Graham BL (2014). Role of spirometry in primary care. Can Fam Physician.

[2] Miller MR, Hankinson J, Brusasco V, Burgos F, Casaburi R, Coates A, Crapo R, Enright P, Grinten CPMVD, Gustafsson P, Jensen R, Johnson DC, MacIntyre N, McKay R, Navajas D, Pedersen OF, Pellegrino R, Viegi G, Wanger J (2005). Standardisation of spirometry. Eur Respir J.

[3] Owens MW, Anderson WM, George RB (1991). Indications for spirometry in outpatients with respiratory disease. Chest.

[4] Ranu H, Wilde M, Madden B (2011). Pulmonary function tests. Ulster Med J.

[5] Cherniak RM, Brown E (1965). A simple method for measuring total respiratory compliance; normal values for males. J Appl Physiol.

[6] Galetke W, Feier C, Muth T, Ruehle K-H, Borsch-Galetke E, Randerath W (2007). Reference values for dynamic and static pulmonary compliance in men. Respir Med.

[7] Desai JP, Moustarah F (2019). Pulmonary compliance.

[8] Ward J (2005). Physiology of breathing. I. Surgery.

[9] Guo YF, Herrmann F, Michel JP, Janssens JP (2005). Normal values for respiratory resistance using forced oscillation in subjects > 65 years old. Eur Respir J.

[10] Bates JHT. Lung mechanics: an inverse modeling approach. Cambridge University Press, Leiden. OCLC: 609842956. 2009. http://public.eblib.com/choice/publicfullrecord.aspx?p=451959 Accessed 2016-07-08

[11] Chiew YS, Pretty C, Docherty PD, Lambermont B, Shaw GM, Desaive T, Chase JG (2015). Time-varying respiratory system elastance: a physiological model for patients who are spontaneously breathing. Plos ONE.

[12] Chiew YS, Chase JG, Shaw GM, Sundaresan A, Desaive T (2011). Model-based PEEP optimisation in mechanical ventilation. BioMed Eng OnLine.

[13] Morton SE, Knopp JL, Chase JG, Docherty PD, Howe SL, Shaw GM, Tawhai M (2018). Development of a predictive pulmonary elastance model to describe lung mechanics throughout recruitment manoeuvres. IFAC-PapersOnLine.

[14] Morton SE, Dickson JL, Chase JG, Docherty PD, Howe SL, Shaw GM, Tawhai M (2018). Basis function identification of lung mechanics in mechanical ventilation for predicting outcomes of therapy changes: a first virtual patient. IFAC-PapersOnLine.

[15] van Drunen EJ, Chiew YS, Chase JG, Shaw GM, Lambermont B, Janssen N, Damanhuri NS, Desaive T (2013). Expiratory model-based method to monitor ARDS disease state. Biomed Eng OnLine.

[16] Panagou P, Kottakis I, Tzouvelekis A, Anevlavis S, Bouros D (2004). Use of interrupter technique in assessment of bronchial responsiveness in normal subjects. BMC Pulmon Med.

[17] Docherty PD, Chase JG, Lotz TF, Desaive T (2011). A graphical method for practical and informative identifiability analyses of physiological models: a case study of insulin kinetics and sensitivity. Biomed Eng OnLine.

[18] Möller K, Zhao Z, Stahl C, Schumann S, Guttmann J. On the separate determination of lung mechanics in in-and expiration. In: 4th European conference of the international federation for medical and biological engineering. Berlin: Springer; 2009. p. 2049–52. 10.1007/978-3-540-89208-3_488. Accessed 08 July 2016.

[19] Howe SL, Chase JG, Redmond DP, Morton SE, Kim KT, Pretty C, Shaw GM, Tawhai MH, Desaive T (2020). Inspiratory respiratory mechanics estimation by using expiratory data for reverse-triggered breathing cycles. Comput Methods Progr Biomed.

[20] Chan EY-T (2007). Use of the interrupter technique in assessment of lung function. J Paediatr Respirol Crit Care.

